# Translation and interpreting services must be considered across the lifecycle of clinical trials

**DOI:** 10.1186/s13063-026-09840-y

**Published:** 2026-06-11

**Authors:** Kirsty Roberts, Rebecca Lewis, Gulnaz Iqbal, Caroline Rick, Victoria Shepherd

**Affiliations:** 1 Bristol Trials Centre, Bristol Medical School, University of Bristol, Bristol, UK; 2 Clinical Trials and Statistics Unit, The Institute of Cancer Research, London, UK; 3 Warwick Clinical Trials Unit, University of Warwick, Coventry, UK; 4 Nottingham Clinical Trials Unit, University of Nottingham, Nottingham, UK; 5 Centre for Trials Research, Cardiff University, Cardiff, UK

**Keywords:** Clinical trials, Inclusion, Limited English proficiency, Translation, Interpreters, Language

## Abstract

**Background:**

Inclusive clinical research requires that all prospective participants are provided with accessible, comprehensible information to support informed decision-making regarding trial participation. However, people with limited English proficiency (LEP) remain disproportionately excluded from trials in English-speaking countries, largely due to insufficient translation and interpretation support. This exclusion compromises equity, participant safety, and the integrity and generalisability of trial findings.

**Main body:**

This commentary highlights why language support must be considered across the entire clinical trial lifecycle, rather than limited to recruitment and consent stages. We examine challenges including identifying which languages and materials require translation; quality assurance of translations; budgeting complexities; reliance on underqualified interpreters; and variability in provision across sites. Practical implications span recruitment, informed consent, outcome measures, intervention delivery, monitoring recruitment and retention, safety reporting, and communication to participants throughout the trial. We emphasise the need for early planning, transparent budgeting, robust quality standards, and researcher training. Drawing on existing frameworks and recent evidence, we provide recommendations on how sponsors, funders, healthcare providers, research teams, and research governance organisations can move towards more systematic and equitable approaches to translation and interpretation.

**Conclusion:**

Addressing linguistic diversity in clinical trials requires a coordinated, lifecycle-wide strategy that goes beyond providing translated materials at the recruitment consultation. System-level changes, such as clear national standards, centralised models of provision, and better training are essential to reduce inequities and enhance the reliability of trial outcomes. Ensuring that people with LEP are supported throughout their participation will strengthen trust, improve safety, and contribute to more representative evidence for healthcare decision-making.

## Background

The ability to make an informed decision about whether to participate in a clinical trial is fundamental to ethical research. Informed decision-making relies on clear, accessible, and appropriately tailored communication. This includes providing information in various formats and delivery methods and adapting materials to meet the diverse needs of the population. Effective communication not only underpins informed consent but also ensures equitable access, participant safety, and the integrity and generalisability of research findings.

However, individuals with limited English proficiency (LEP) living in English-speaking countries such as the UK are often excluded from clinical trials due to inadequate language support [1]. People with LEP may face additional challenges, including low literacy in their first language, limited health literacy, and intersecting factors such as age and/or socio-economic status which can vary across different communities [2]. One common approach to improving inclusivity is to translate study documents into other languages and provide interpreters during research consultations. Whilst these steps are important, offering selected information in different languages only partially addresses the complexity of language-related barriers and is just one component of a wider and more comprehensive strategy which is required for inclusive research participation. Language is not simply a vehicle for transferring information, it plays a crucial role in building trust, helping individuals feel welcome and valued, and ensuring fully informed consent. The appropriate approach to translation needs to be considered, either using literal translation, maintaining the content and language form of the original text ‘word for word’ or using functionalist approach in which translators consider the purpose of the original text and adapt it to the needs of the target audience. However, providing written translations on their own is usually not sufficient as this can lead to different understanding between different languages and cultures [3]. If this occurs, translation can in fact hinder research participation rather than support inclusive participation. This highlights the need for translation and interpretation to be conducted to a high standard using robust and culturally appropriate methods at all points of contact. However, achieving this can be challenging, time-consuming and costly. These demands must also be balanced proportionately, as for example, recent census data indicate that only 1.6% of the UK population speaks little or no English [4]. Consequently, even comprehensive language provision may address only a subset of the barriers faced by underserved groups when participating in clinical trials.

In clinical trials, the individuals responsible for planning, budgeting, and delivering language support services are often not multilingual and likely lack formal training in linguistics. Although some guidance exists on the use of translators and interpreters in health and social care research [5, 6], there is limited training for professionals in this area and no national standards to guide best practice. Similar concerns have been reflected in NHS healthcare settings. A recent NHS England framework report described community language services as inconsistent and called for national standards to provide high-quality translation and interpreting for people with LEP [7]. It also remains unclear to what extent existing guidance is being implemented, or how effective it is in practice. The ACCESS study, which explored strategies used by clinical trials units (CTUs) to support inclusive research, highlighted key challenges in implementation and a lack of consistent standards for language support. Whilst translation services were frequently included in research budgets, little data exist on how often these services were used or how successful they were in facilitating trial participation [8]. Moreover, language support efforts tend to focus on the early stages of trial conduct, such as providing translated participant information leaflets (PILs) and interpreters at the recruitment stage, rather than being maintained across the entire research lifecycle.

Language barriers can significantly affect both the interpretation and generalisability of trial results. This has been particularly evident in studies of long COVID which highlighted issues of misinterpretation of outcome measures from people from different cultures [9]. There is limited evidence on how language proficiency impacts participant retention and safety, likely due to the difficulty of systematically capturing such data during the trial process. Currently, published guidance [5, 6] remains the primary resource available to researchers, yet significant knowledge gaps persist regarding the effectiveness of recommendations and whether they are followed. These gaps may lead to inefficiencies and pose risks to participant safety, particularly in trials involving new interventions or clinical trials of investigational medicinal products (CTIMPs).

In this paper, we examine the importance of providing language support across all phases of the trial process. We outline key considerations at different stages of the trial lifecycle, explore the practical challenges of implementing language services within the UK National Health Service (NHS) context and across diverse trial settings, and offer recommendations to support stakeholders. Finally, we identify priorities for future methodological research, infrastructure development, and training in this area.

## Main text

### Translation and interpreting services

#### Recruitment and informed consent

Restrictive eligibility criteria, such as narrow consent and language requirements, have disproportionately excluded individuals from underserved groups in clinical trials [10–12]. There needs to be a clear justification to only include individuals who are proficient in English, such as an intervention involving behavioural group therapy which necessitates delivery in a sole language (mitigations discussed later). Eliminating these criteria is now actively encouraged, enabling inclusion of participants who speak languages other than English, but this requires the provision of translated trial documents and/or interpretation services. Knowing which languages to translate documents into and ensuring that translated documents are understandable to participants can be a significant challenge [13].

It is also important to recognise that trial documents themselves are only one part of the consent process. Under Part 3 of Schedule 1 to the Clinical Trial Regulations, the informed consent process should involve ‘an interview with the investigator, or another member of the investigating team, in which he has been given the opportunity to understand the objectives, risks and inconveniences of the trial and the conditions under which it is to be conducted’ [14]. For example, the latter criterion includes transparency, and the potential participant should be made aware during the consent process as to whether patient-reported outcome (PRO) measures are available in formats other than written English in the case of pragmatic clinical trials. Previous studies have concluded that to improve understanding, the focus should be directed more towards the communication between the person responsible for obtaining consent and the potential participant [15]. Interpreters can support communication between researchers and participants when they do not speak the same language. In the UK, the NHS often provides interpretation for patients via external telephone services such as ‘Language Line’ which has audio interpretation for more than 200 languages or via their own in-house interpretation services, however, access to interpretation is inconsistent across the NHS. Additionally, the relationship between the person seeking consent and the potential participant is viewed as an essential component of the informed consent process. Informed consent is more than just ‘getting a signature’ and relationality, reciprocity, and trust are key [16]. This is particularly relevant when approaching people from underserved groups where trust is frequently cited as a barrier to inclusion and can be negatively impacted by a lack of cultural and linguistic awareness amongst research staff [1, 17]. It can be challenging for staff approaching potential participants to pick up on subtle linguistic ‘clues’ in language which may indicate misunderstanding or a lack of comprehension on the part of the potential participant especially when these cues are nuanced or ‘lost in translation’. This may also affect determining eligibility for a clinical trial when cultural or linguistic differences influence how symptoms such as pain or distress are described or interpreted. Screening for clinical trial eligibility may occur over multiple consultations, with earlier clinical encounters often used to gather preliminary information. Briefing interpreters before consultations with people with LEP is considered good practice to ensure the trial is explained accurately [6]. Where multiple encounters are required, it is preferable to use the same interpreter, as they will already be familiar with the study context; however, this is often challenging due to limited interpreter availability on site.

Another barrier reported by researchers is the absence of a standardised written form of a participant’s spoken language. For example, people from the Sylhet region of Bangladesh, whose primary language is Sylheti, and those from the Pakistan-administered region of Azad Kashmir, whose main language is Mirpuri, made up more than 50% of patients in the outpatient department of a UK hospital participating in a diabetes trial. Both spoken languages have no written form [18]. In these situations, alternative approaches to consent could include creating audio-recordings of the participant information sheets and recording participants’ verbal consent [18].

Some participants with LEP may have additional communication or decision-making needs due to a condition such as stroke or dementia. Supporting participants to provide their own consent wherever possible is a key principle of mental capacity legislation (e.g. Mental Capacity Act 2005 [19]). This intersectionality may present additional barriers to inclusion as information needs to be provided in an accessible form and in a language that is used by the participant. Examples of strategies to support inclusion, as reported by authors of a scoping review on including people from ethnic minorities in dementia research, include the use of interpreters and the translation of study materials, followed by the employment of bilingual and bicultural researchers, and the use of flexible modes of participation that take into account different levels of literacy [20].

Decisions as to whether someone is proficient in English, where required for trial participation, are typically carried out by research staff who are not linguistic experts, often with no formal assessment process. Biased viewpoints or lack of knowledge on the part of the research staff may lead to potential harm due to eligible patients being deemed as not having ‘good enough English’ to be invited to join the study. There should be explicit guidance as to how this assessment will be made during the recruitment process, warranting further methodical research in this area.

In all studies, consent should be an ongoing process and participants’ willingness to continue to participate should be reaffirmed throughout the study. Participants also have the right to change participation at any time during a clinical trial, and it is sometimes necessary to seek re-consent from participants where there is a material change to the study design. This means that there are ongoing requirements to ensure that participants with LEP can reaffirm their ongoing participation, be re-consented if necessary, or withdraw if they wish at any point. This may require ongoing access to translation and interpretation services which can bring logistical challenges where those services have limited provision.

#### Outcome measures

The UK National Institute for Health and Care Research (NIHR) encourages the use of outcome measures that reflect patient experience, beyond clinical metrics. This has led to the development and validation of PROs for pragmatic RCTs, which capture individuals’ perceptions of their health, well-being, and daily functioning. Such measures enable evaluation of interventions in terms of their impact on quality of life, aligning with NHS values. Current practice where many PROs are both created and validated only in written English may lead to the questionnaires not reflecting the items most important to people from underserved groups. How PROs are measured may only reflect the views and experiences of English-speaking groups and lead to individuals with LEP withdrawing or dropping out from trial participation [21](see below). Furthermore, it may lead to less reliable data being reported if participants rely on friends, family [6] or non-validated technologies to translate for them e.g. Google Translate. Where friends or family members are translating there is a further concern that participants may modify their answers to be more acceptable to their loved ones; therefore, sponsors often explicitly instruct sites not to do this.

Significant investment is needed to develop robust, culturally sensitive PROs that, ideally, are validated in multiple formats including written and oral versions. The translation process should focus on achieving conceptual equivalence, making sure the original meaning is accurately conveyed in the target language. This could be rationalised by focussing on PROs that are the primary outcome measure to ensure that these data are as complete and representative as possible. However, this solution does increase the likelihood that the population responding to the primary outcome may not be the same as that for the other outcome measures in a trial. Focussing on PROs that are part of core outcome sets (COMET Initiative) [22], which, in turn, could reinforce the use of core outcome set measures making it easier to meta-analyse inclusive datasets in future and could broaden the information from the whole population.

It is, however, not generally feasible to conduct translation and revalidation of PROs or other outcome measures into multiple languages during study setup. For PROs, researchers could consider whether using validated instruments already available in other languages may be appropriate in their projects, such as the Quality-of-Life questionnaires EQ-5D and EORTC, both of which have a robust translation and validation process [23]. Where participants have low literacy in their first language, trained staff or interpreters may provide support during questionnaire completion; the EQ-5D, for example, is available in an interviewer-administered mode [23]. Lessons could also be learned from strategies developed for other groups struggling with written English such as people with aphasia, that could be incorporated into improving communication with people who primarily communicate in languages other than English [24]. For pragmatic clinical trials, developing new PROs in multiple languages simultaneously would help decentre the process and reduce the risk of cultural bias [25].

#### Monitoring recruitment and retention

Inclusive research design, now a condition of NIHR funding, not only requires the inclusion of underserved groups and strategies to improve their participation, but also the monitoring of the success of those strategies during the lifecycle of the project, with further action taken if strategies are ineffective. Monitoring the uptake of translations and use of interpreters will allow an assessment of their utility. However, remediation strategies targeting individuals with LEP may require additional time, funding, ethical approval, and specialist input. Their implementation and evaluation are often constrained by tight trial timelines and funding constraints.

Monitoring any differential rates of withdrawal of participants with LEP and the reasons for withdrawal (or other changes to participation status) is also important to understand how representative the trial population is of the target clinical population. To aid the assessment of whether trial results reflect the consented population, it is critical to collect and review demographic data about participants throughout the trial [26]. This is recommended both after trial entry, at the primary outcome measure and other pertinent endpoints, and to monitor use of language support interventions. This will help to identify whether the voices of people with LEP are being selectively lost and ideally the reasons why. There is likely to be intersectionality between people with LEP and other underserved groups, so monitoring is essential for insight into whether people genuinely have equal access to participate in research.

#### Interventions

Engaging in clinical trials can present multiple barriers for participants with LEP, particularly when the intervention involves online or digitally delivered therapies. These may be difficult to access which can lead to withdrawal from the trial or the intervention appearing to underperform in certain populations. This may occur if it is not adequately described or culturally sensitive aspects have not been considered during translation. This issue can be compounded by digital exclusion which can be a significant problem particularly where there is intersectionality between language, socio-economic status and older age.

Ensuring equitable access to behavioural therapy interventions for people with LEP also poses challenges, particularly if there are time-constraints (e.g. delivery within a limited window from surgery) or the intervention is delivered in a group setting. These difficulties are compounded by a lack of research-active therapists in the UK who can deliver the intervention in languages other than English. The ability to tailor interventions for people with LEP may be further restricted by copyright holders, who may have mandated strict rules for delivery of the intervention including translation or the use of interpreters. This challenge has been recognised by the NIHR and groups investigating behavioural therapy interventions that were limited to individuals who spoke English have been funded with the proviso that a parallel work stream or Study Within a Trial (SWAT) investigates how the intervention can be adapted and/or implemented for people with LEP [27, 28].

In CTIMPs, additional barriers arise when participants with LEP are expected to self-administer study medications. To ensure safe and effective participation, translated labelling should be considered including clear instructions on dosage (how many, how often), administration (before or after food), warnings (may cause drowsiness, information on drug interactions) and storage information (refrigerate if required). These aspects should be considered during study setup and discussed with any pharmaceutical partners providing the IMP. Where multinational companies are involved, they may already have translated package inserts or labelling in other languages. If providing labelled products, arrangements for translated labels should be covered in drug supply contracts.

#### Safety reporting

Language barriers in clinical trials can significantly affect the accurate reporting, detection, and interpretation of adverse events (AEs). Evidence suggests that toxicity profiles may vary across ethnic groups; when language barriers are present, the reporting of such events may be further compromised [29, 30]. This has important implications for participant safety, the integrity of trial data, and compliance with regulatory standards.

Participants with LEP, without sufficient support, may struggle to articulate symptoms or understand when and how to report AEs, resulting in under-reporting from certain groups. Miscommunication may also lead to delays in recognising clinically significant AEs or a misclassification of their severity [31]. Patient diaries are often used to capture safety data between study visits. If diaries are only provided in English, participants with LEP may be unable to use them effectively. As with informed consent materials, data collection tools should be professionally translated into the participant’s preferred language to ensure completeness and accuracy [3, 32, 33]. This is particularly important where patient-reported outcomes play a role in safety monitoring.

Where interpreters are used, cultural differences in the expression of illness, differences in health beliefs, and understanding of medical terms can compromise the accuracy of symptom reporting. Symptoms may be described using culturally specific idioms or metaphors, making them difficult to translate or interpret accurately, particularly in a busy clinical setting [34]. Furthermore, interpreters may unintentionally filter or modify participant statements [35, 36].

Power dynamics between research staff and participants may also contribute to the under-reporting of AEs. In clinical trial settings, participants with LEP may feel disempowered to question procedures or report discomfort, particularly if they fear negative consequences such as reduced access to care or withdrawal from the study [37]. Improving AE reporting among participants with LEP requires trials to proactively consider linguistic diversity, provision of translated materials, culturally competent staff training, and the involvement of bilingual researchers.

#### Withdrawal/loss to follow-up (FU)/missing data

Individuals with LEP may be more likely to withdraw from or reduce participation in clinical trials, potentially compromising the applicability of findings [38]. However, trials rarely report withdrawal reasons related to language barriers, making the extent of this issue difficult to quantify. For instance, participants with LEP may be misclassified as lost to follow-up rather than having actively withdrawn. Trial designs may also introduce differential data collection, depending on factors such as data source (clinical vs. patient-reported), outcome prioritisation (primary vs. secondary), or follow-up schedules. These variations can result in incomplete data for certain outcomes among LEP participants. For example, clinical data may be available while PROs are missing.

Return rates by ethnicity are seldom reported, and even when available, ethnicity is not a reliable proxy for LEP status [39]. Consequently, disparities in data completeness may go undetected except through individual patient-level meta-analyses that include both ethnicity and language proficiency. Such differential data collection may distort the perceived effectiveness of interventions for LEP communities. For example, if survival data are available but PROMs are missing, future patients from these communities may have clinical decisions made about them based on incomplete evidence, either relying solely on survival metrics or on quality-of-life data that do not reflect their experiences.

Clinical trials rely on the complete and accurate collection of data, including information related to participants changing, reducing or stopping their participation. Whilst much attention has been given to enrolment and informed consent, significantly less attention has been placed on language support at the point of withdrawal. Trials may lack interpreters or translated materials at the time of withdrawal, leaving participants without the tools needed to document their experiences effectively, resulting in missing, incomplete or inaccurate data, which can introduce uncertainty and mask safety issues. Staff may document vague reasons or misinterpret the participants’ reasons or participants simply stop attending appointments and are classified as ‘lost to follow-up’.

#### Communication with participants

Effective and inclusive communication strategies with trial participants and the broader community are a vital component of trial design and should be embedded at the proposal development stage. A dedicated communication plan should be clearly described in ethics applications, fully costed within funding proposals to ensure accessible communication, reviewed, and updated throughout the trial lifecycle. The plan should outline how participants will be informed and engaged throughout the study, including methods for sharing study updates, protocol changes, and final results. Despite its importance, many trials fail to communicate results to participants due to limited planning, inadequate guidance, and insufficient resources [40, 41]. Ensuring timely, understandable study updates and end-of-study summaries through newsletters, text messages, or digital platforms for participants with LEP will strengthen trust and support participant retention.

For participants with LEP, inclusive communication requires specific and proactive measures. These include budgeting for the translation of all participant-facing materials, interpreter services to support informed consent, re-consent following protocol amendments, ongoing check-in calls, data collection, and in-person visits. Participant language preferences should be recorded in trial database systems to enable consistent, tailored communication throughout the study. These measures reduce attrition and promote inclusive engagement across diverse populations [8], as well as enhancing the participant experience and contributing to the ethical conduct of trials. New HRA guidance mandates the dissemination of understandable results of CTIMPs within 1 year of study completion, which will come into effect in April 2026 [42].

Building diverse research teams, across language, ethnicity and background is needed for delivering inclusive and equitable health research. Multilingual staff play a critical role in making research more inclusive and accessible to patients and communities with LEP. Their involvement can enhance trust, improve communication, and reduce barriers to participation, particularly in underserved populations that are historically underrepresented in research. Linguistically diverse staff can also aid informed consent, facilitate culturally sensitive engagement, and support more representative recruitment and retention [37].

### Funding and budgeting for translation and interpretation services

#### Early planning and estimating language needs

Planning and budgeting for translation and interpretation services is often complex and underdeveloped at the grant application stage. At the pre-award stage, trial teams need to estimate how many languages to translate into, which materials will require translation, and in what formats (e.g. written, audio, video) based on their trial population. These decisions are often hindered by uncertainty regarding site selection and participant demographics. Tools such as the INCLUDE Framework, Trial Forge 3 guidance and STRIDE recommendations provide helpful starting points for identifying under-served populations and relevant language needs [5, 43–45]. Engagement with community groups with lived experience of the condition can offer insights into local linguistic and cultural diversity where projects are running in selected areas. Disease prevalence data, census information, and consultation with healthcare staff may further inform which languages to prioritise [5].

In multinational studies, estimating language needs across yet-to-be-confirmed sites is particularly challenging. Some teams budget for a maximum number of languages, using average translation costs (which vary by language rarity), but this approach may not reflect actual need and risks excluding smaller language communities. Where feasible, surveying potential sites about common languages spoken can improve accuracy, although this may not be possible given limited timelines and resources.

#### Central vs local provision of translation services and approvals

The guidance for submitting applications for regulatory approvals in the UK [46]offers ambiguous advice regarding the responsibility for translation services [47]. Chief Investigators are instructed to ensure site-level arrangements are made by local investigators for their non-English-speaking patients (IRAS Q A33-1). However, it also suggests that there could be centralised commissioning of translations to ensure cost-effectiveness and consistency. There is also a lack of clarity over central Research Ethics Committee (REC) and Health Research Authority (HRA) oversight of translated materials, with no requirement to submit patient-facing materials in languages other than English for review or on how to document use of interpreters in the informed consent process [48]. These contradictions contribute to uncertainty around where responsibility, costs and quality control should lie.

A practical solution may be for sites to provide translation and interpreting services based on their local populations and charge research teams accordingly. This decentralised model supports tailored provision but requires careful communication and planning at the grant stage to avoid duplication or gaps in service.

#### Quality assurance of translations

Translation quality is essential to ensure that research documents are accurate, accessible, and culturally appropriate. However, this is often overlooked in budgeting. Reputable providers should follow international standards (ISO 17100:2015) but trial teams should seek clarity regarding their translation approaches and standards. Back translation, re-translating a document into the original language for verification, is a widely recommended practice to ensure conceptual equivalence [5, 6]. Evidence suggests that literal translation results in more errors and misunderstandings, whereas functionalist approaches are more effective for participant comprehension [3].

Where possible, translation should involve multiple professional translators, clinical review, readability checks and end-user testing [49]. Involving community members in back translation or end-user testing also fosters trust and enhances cultural relevance. They can also play a key role in tailoring PRO measures, particularly when terms lack direct equivalents or carry different connotations across languages [9] However, community-based translation services are rarely offered as part of a comprehensive professional package.

#### Estimating translation costs

Translation is typically costed according to language rarity and cost per word, requiring teams to estimate word counts of documents before they are finalised, which is an inherently difficult task during grant development. While some CTUs use estimates from previous studies (e.g. Patient Information Leaflets and consent forms), other trial-specific documents, such as PROs, diaries, and patient safety reporting materials, are harder to estimate. Any amendments made to trial documents during the trial will incur additional costs; therefore, provisions need to be made to budget for changes in any of the translations. Multimedia formats (e.g. videos, voiceovers, or subtitles) often require different suppliers and separate costings. This further complicates budgeting, highlighting the need for institutional frameworks or support systems to streamline procurement. Institutions with high research volumes might consider establishing framework agreements with preferred translation providers. This model enables cost sharing across projects, ensures consistent quality, and coverage of additional translation needs during amendments or dissemination (e.g. lay summaries).

#### Budgeting for interpretation services

Interpretation services, unlike translation, are generally costed by time (per hour or minute), with rates influenced by interpreter qualifications, language rarity, and format (in-person vs remote). The NIHR recommends multiplying the estimated number of hours by 2.5 to accommodate the longer time required for interpretation. Additional costs may also include interpreter travel for in-person consultations, which some providers may not cost in or raise with trial teams as the travel times are mostly unknown at this time [6].

Research teams may need to budget for the additional staff time involved in interpreted consultations. In the UK, additional time for NHS site staff can be recorded in the Schedule of Events Cost Attribution Tool (SoECAT) which forms part of the UK’s essential document set when obtaining trial approvals. These can be included as Service Support Costs if used for recruitment or consent or Treatment Costs if the interpreted consultation is part of the treatment pathway. It is good practice for NHS site staff to brief interpreters prior to sessions to ensure cultural nuances are understood and the trial is explained correctly [6]. Whether this briefing time is included in the providers’ contracts, or needs to be costed separately, varies and should be clarified early.

In NHS settings, another complexity is the preference of some sites to use their own interpreting services. These preferences often only emerge at the site set-up stage, making accurate early budgeting difficult. Site surveys, as previously described during grant preparation, could help anticipate this, although not definitively, as the final site group may change upon delivering the trial, including the addition of more sites if there are delays to recruitment. An alternative model is to delegate the responsibility for interpretation entirely to NHS sites and clearly document this in the SoECAT. While this may lead to variation in provision, central trial teams could provide standardised guidance and training on expectations around interpretation.

#### Systemic limitations of using interpreters in an NHS setting

Current NHS costing models are insufficiently flexible to support the time-sensitive demands of coordinating interpreters, particularly for consent processes during clinical appointments. They can also fail to account for cultural sensitivity and patients’ preferences when discussing intimate personal issues, for example regarding the gender of the interpreter or having the same person act as interpreter at multiple appointments to facilitate understanding and avoid having to repeat the same information multiple times [7, 50]. Costing models need to account for both the amount and timing of staff input, especially when interpreters must be arranged at short notice. Quality assurance also remains a concern. Although the use of interpreters with a Level 6 Diploma in Public Service Interpreting is recommended [6], this is not mandatory which will lead to variation in interpreter skill and experience. Innovative models such as the LASI initiative [51], offering video-based, on-demand, easy-access professional interpretation, could provide an alternative. These systems, when supported by trial-specific training and ethically approved documentation, could be implemented at regional or national levels, and a centralised service of trained interpreters for portfolio supported-funded trials could be recruited.

### Training, support and future research

In a cross-sectional survey of 339 researchers in the United States, the most frequently cited barriers to including individuals who speak languages other than English were a lack of training on inclusive research practices and the predominance of English-only speaking research staff [52]. To address these challenges, 73.5% of respondents recommended training on interpretation and translation services, while 67.6% suggested that networking with researchers experienced in working with linguistically diverse populations would be beneficial. Although partnerships with community-based organisations were infrequently reported, those who engaged in such collaborations found them advantageous. Additional recommendations included improving awareness of locally spoken languages (49.3%) and receiving support in recruiting bilingual staff (39.8%). Applying these recommendations to UK clinical trials may, however, be more complex due to significant differences in the linguistic landscape. Unlike the United States, where Spanish represents a large, widely spoken second language population, no single non-English language accounts for a similar proportion of speakers in the UK.

In the UK context, findings from the ACCESS study, which involved 40 trial experts, emphasised the importance of enhancing researcher training to facilitate the design of more inclusive clinical trials [8]. The study also revealed a lack of standardised processes for translation and interpretation in clinical trials which was identified as a significant barrier to inclusive research. Existing organisations such as English Unlocked and the Centre for Ethnic Health Research (CEHR) offer targeted training for researchers seeking to improve their understanding and implementation of language services in trials. For instanceEnglish Unlocked provides a 2 -h workshop on effective collaboration with interpreters [53], while CEHR delivers cultural competency training which would support and enhance researchers’ awareness of participants’ rights to accessible language information, and the importance of cultural sensitivity [54]. However, careful planning would be required to determine the provision of this training.

Within the UK health and social care setting, a recent survey of 190 NHS sites revealed considerable variability in staff knowledge regarding language support. Only 46% of respondents were aware of their local Trust’s policies on interpretation and translation, and 16% reported being unaware of the languages commonly spoken in their local area [55]. This inconsistency has been recognised separately by the NHS who have now introduced a new framework [7]. If implemented nationally, this framework could provide a valuable resource for researchers conducting clinical trials within NHS settings, enabling more consistent and higher-quality translation and interpretation services. Nevertheless, ambiguity remains regarding whether responsibility for language support lies with the NHS or the host research institution, necessitating further clarification. While several commercial providers offer comprehensive and tailored language services, there is increasing interest in establishing a centralised service hosted within the NIHR infrastructure or in partnership with NHS England to promote equitable inclusion in research. Such a service could standardise translation quality and regulatory compliance, reduce costs for individual trial teams, enhance recruitment and retention of diverse participants, and support data harmonisation across multi-site studies.

The use of artificial intelligence (AI) to support text translation, commonly referred to as machine translation (MT) and often using large language models (LLM) is becoming more prevalent among the public and within healthcare contexts, with platforms such as Google Translate, Bing Translate, and DeepL being widely used [56]. While MT offers convenience and efficiency, its application in research should be approached with caution due to concerns about accuracy and potential implications for patient safety. By their nature, LLMs are susceptible to ‘hallucinations’ where returned results are not accurate, so any AI-generated translations should undergo back translation (by a different system or by human interpretation) [57]. As with non-AI translations, they should also ideally be reviewed for cultural sensitivity by people fluent in the relevant language and/ or with lived experience, and should adhere to any relevant national ethical guidelines; however, these are yet to be established within the UK healthcare setting [7]. It is essential that researchers must therefore exercise a risk-proportional approach, with early evaluation ideally conducted in low-risk studies before integration into larger and more complex trials. It must also be recognised that AI has the potential to act as both a facilitator and a source of inequity. Individuals with LEP are disproportionately represented in the most deprived 10% of neighbourhoods in England and are consequently at greater risk of digital exclusion [7]. They, therefore, may be further excluded if they are required to access the MT themselves. Although MT could help translate PILs and other written materials, some groups are unable to read or interpret written text, meaning alternative modes such as MT-generated videos (which create voiceover and video from written text using text-to-speech technology) may be necessary [58].

Overall, MT technologies offer substantial potential to improve efficiency and expand access to language support in clinical research. When rigorously validated and implemented with appropriate oversight, they can enhance inclusivity by delivering timely, cost-effective translations for participants who may otherwise be excluded. However, without adequate safeguards, quality controls, and attention to equity, MT risks compromising participant safety or reinforcing existing disparities. A balanced approach is therefore essential, one that recognises the value of MT while ensuring proportionate risk management, cultural sensitivity, and ongoing human oversight. With such measures in place, progress rather than perfection can guide the responsible integration of MT into clinical research.

The recommendations proposed in this paper to enhance translation and interpreting throughout the clinical trial lifecycle will inevitably introduce additional costs and time requirements. It is therefore essential that:

1. Innovative approaches are rigorously evaluated—for example, through SWATs or smaller methodological projects conducted across diverse trial populations and settings to ensure their effectiveness.
2. Resources are widely disseminated, easily accessible, and shared wherever possible, such as via repositories [59, 60]and online platforms like the Explain initiative [61]and MAPLE website and associated PIL [62, 63].
3. Methodological evaluations of clinical trials should report expected and actual participant demographic characteristics [64] to direct sufficient resources towards equitable language support provision.

In the ACCESS study, translation was identified as a key area for evaluation. The authors recommended surveying CTUs to understand current practices in language service provision and to assess their impact on the recruitment of ethnic minority participants [8]. In response, the INVITE study [65] was developed to assess translation and interpreting services across multiple trials using a mixed-methods approach. Findings from this study are expected to be disseminated by early 2027.

Once these strategies are supported by a stronger evidence base, they may be more efficiently implemented through shared models, across services, regions, or even nationally rather than on a trial-by-trial basis. This further strengthens the case for a centralised model of language provision in healthcare research, underpinned by robust infrastructure and coordination.

## Recommendations

Ensuring there is appropriate language support for potential trial participants across all phases of the trial process requires a system-wide approach involving sponsors, funders, employing organisations, healthcare providers, researchers, and research governance organisations. In Table 1, we propose a series of recommendations for stakeholders to improve the current support provided for people with LEP to ensure equitable access, informed consent and participant safety, and therefore ensure the integrity and generalisability of research findings.

**Table 1:** Recommendations for improving language support and inclusion of people with limited English proficiency (LEP) in clinical trials

	Key stakeholders*				
	Funders	Regulatory bodies (e.g. HRA)	Sponsors	Trial teams/researchers	Participating sites (NHS/social care/community settings)
Guidance, resources and support					
Clear guidance on translation and interpretation provision in trials, including minimum acceptable standards for professional interpreters and translation services (e.g. Level 6 Diploma in Public Service Interpreting and ISO 17100:2015)		✓			
In UK setting, consider setting up central resource or service to support interpretation/translation for non-commercial portfolio trials	✓	✓			
Policies for translations and interpretation in research, including where translated materials are not available			✓		
Trial logistics					
Ensure translations and interpretations are considered and adequately costed for the whole trial lifecycle, including: • Trial entry/informed consent • Intervention delivery (e.g. IMP labelling/leaflets, behavioural interventions) • Endpoint data capture including patient reported outcomes (PROs) • Safety monitoring • Ongoing verification of consent/reconsent (as needed) • Communication with participants • Dissemination of results • Amendments to any of the above	✓			✓	
Ensure appropriate languages for translations are identified during trial setup (e.g. through site surveys, routine census data, public and patient involvement and engagement (PPIE), community groups)				✓	✓
Exercise caution if using AI for translation—consider known issues with large language models (e.g. hallucinations) and introduce mitigation			✓	✓	
Implement flexible modes of participation that can account for different levels of literacy and language proficiency				✓	
Consider involving bilingual research staff/ PPIE advisors or local community groups to proofread translated materials and advise on cultural validity				✓	
Carefully consider whether to arrange translation/interpretation for PROs, recognising the need for a robust and validated process. Consider using existing validated translated PRO instruments where available				✓	
Ensure availability of interpreters for each trial-related appointment				✓	✓
Do not rely on multilingual friends, family members or (non-interpreter) staff to provide formal interpretation for trial enrolment or follow-up visits				✓	✓
Training					
Training on general considerations around language, including cultural competency, identification of patients requiring communication and/or language support, and local policies/support for translations and interpretations			✓	✓	✓
Train sites on expectations around information giving and use of translated materials/interpreters for each trial				✓	
Data and reporting					
Record and report need for communication and/or language support and use of translated materials/interpretation				✓	✓
Record and report trial participants reducing/stopping participation due to language barriers				✓	✓
Monitor enrolment and drop out by preferred language throughout recruitment (including reasons for drop out if possible)				✓	

^*^Recommendations may apply to other stakeholder groups depending on the context

## Conclusions

The inclusion of people who speak a language other than English in clinical trials requires careful planning, including appropriate budgeting for translation and interpretation services which is often both costly and complex. Addressing linguistic diversity involves not only cost estimation, but also collaboration with communities and patient and public advisors, quality assurance processes, and flexible infrastructures. Institutions, funders, and policymakers should support models that prioritise inclusion and feasibility, such as framework agreements, national interpretation services, and updated costing guidance to better serve under-served populations and reduce barriers to participation. Whilst it is not feasible to provide written translations in all languages, sponsors should have a policy about inclusion and ongoing communication with participants where translated materials are not available.

Offering only translations of patient information prior to trial entry is likely insufficient to adequately support ongoing inclusion. Provision of translated materials needs to be considered throughout the entire trial life cycle. We recommend that any researchers providing translated materials or interpretation services collect and report information about their use and monitor their trial populations to assess impact.

## Data Availability

Not applicable.

## Abbreviations

AE: Adverse event
AI: Artificial intelligence
CEHR: Centre for Ethnic Health Research
CTIMP: Clinical trial of investigational medicinal products
CTU: Clinical trials unit
HRA: Health Research Authority
LEP: Limited English proficiency
LLM: Large language models
MT: Machine translation
NHS: National Health Service
NIHR: National Institute for Health and Care Research
PIL: Patient Information Leaflet
PRO: Patient-reported outcomes
REC: Research Ethics Committee
SWAT: Study Within a Trial
SoECAT: Schedule of Events Cost Attribution Tool

## References

[1] Bodicoat DH, et al. Promoting inclusion in clinical trials-a rapid review of the literature and recommendations for action. Trials. 2021;22(1):880. 34863265 10.1186/s13063-021-05849-7 PMC8643184

[2] Ethnicity Facts and Figures: English language skills: By ethnicity and sex. 2018 26 March 2024 Cited 2025 30 September; Available from: https://www.ethnicity-facts-figures.service.gov.uk/uk-population-by-ethnicity/demographics/english-language-skills/latest/#by-ethnicity-and-sex.

[3] Colina S, et al. Research documents for populations with limited English proficiency: translation approaches matter. Ethics Hum Res. 2022;44(1):29–39. 34936237 10.1002/eahr.500115

[4] GOV.UK. English Language Skills. 2018 Cited 2026 24/03/2026; Available from: https://www.ethnicity-facts-figures.service.gov.uk/uk-population-by-ethnicity/demographics/english-language-skills/latest/

[5] Dawson S, et al. Trial Forge Guidance 3: randomised trials and how to recruit and retain individuals from ethnic minority groups-practical guidance to support better practice. Trials. 2022;23(1):672. 35978338 10.1186/s13063-022-06553-w PMC9383663

[6] NIHR ARC North East and North Cumbria: How to conduct research involving interpreters and translators. 2022 10/10/2025]; Available from: https://arc-nenc.nihr.ac.uk/wp-content/uploads/2022/12/Guide-Part-Two-How-to-conduct-research-involving-interpreters-and-translators.pdf.

[7] NHS England: Improvement framework: community language translation and interpreting services. 2025 Cited 2025 23/10/2025; Available from: https://www.england.nhs.uk/long-read/improvement-framework-community-language-translation-and-interpreting-services/.

[8] Biggs K, et al. Time to STEP UP: methods and findings from the development of guidance to help researchers design inclusive clinical trials. BMC Med Res Methodol. 2024;24(1):227. 39358688 10.1186/s12874-024-02342-y PMC11445965

[9] Khunti K, et al. Long COVID research in minority ethnic populations may be lost in translation. Nat Med. 2024;30(9):2390–1. 38918633 10.1038/s41591-024-03070-y

[10] Isaacs T, et al. The inclusion of ethnic minority patients and the role of language in telehealth trials for type 2 diabetes: a systematic review. J Med Internet Res. 2016;18(9):e256. 27670360 10.2196/jmir.6374 PMC5057063

[11] Santos JD, et al. Most UK cardiovascular disease trial protocols feature criteria that exclude ethnic minority participants: a systematic review. J Clin Epidemiol. 2024;167:111259, 111259. 38215800 10.1016/j.jclinepi.2024.111259

[12] Moloney C. Underserved groups remain underserved as eligibility criteria routinely exclude them from breast cancer trials. J Clin Epidemiol. 2022;147:132–41. 35341945 10.1016/j.jclinepi.2022.03.011

[13] Brelsford KM, Ruiz E, Beskow L. Developing informed consent materials for non-English-speaking participants: an analysis of four professional firm translations from English to Spanish. Clin Trials. 2018;15(6):557–66. 30295050 10.1177/1740774518801591 PMC6218315

[14] UK Government: The Medicines for Human Use (Clinical Trials) Regulations 2004 (No. 1031). 2004: www.legislation.gov.uk.

[15] Eeckhout D, Aelbrecht K. Informed consent: research staff’s perspectives and practical recommendations to improve research staff-participant communication. J Empir Res Hum Res Ethics. 2023;18(1–2):3–12. 36562147 10.1177/15562646221146043

[16] Adams M, Caffrey L, McKevitt C. Barriers and opportunities for enhancing patient recruitment and retention in clinical research: findings from an interview study in an NHS academic health science centre. Health Res Policy Syst. 2015;13(1):8, 8. 25971302 10.1186/1478-4505-13-8 PMC4429658

[17] Hughson J. A review of approaches to improve participation of culturally and linguistically diverse populations in clinical trials. Trials. 2016;17(1):263. 27229153 10.1186/s13063-016-1384-3 PMC4880985

[18] Lloyd CE, et al. Securing recruitment and obtaining informed consent in minority ethnic groups in the UK. BMC Health Serv Res. 2008;8(1):68, 68. 18373876 10.1186/1472-6963-8-68 PMC2311303

[19] Mental Capacity Act 2005. 2005.

[20] Brijnath B, et al. Including ethnic minorities in dementia research: recommendations from a scoping review. Alzheimers Dement Transl Res Clin Interv. 2022;8(1):e12222. 10.1002/trc2.12222 PMC9053375 35505899

[21] Rimmer A. Can patients use family members as non-professional interpreters in consultations? BMJ. 2020;368:m447. 32047051 10.1136/bmj.m447

[22] Williamson PR, et al. The COMET handbook: version 1.0. Trials. 2017;18(3):280, 280. 28681707 10.1186/s13063-017-1978-4 PMC5499094

[23] EuroQol Research Foundation: Translation process. 2025 10/10/2025]; Available from: https://euroqol.org/register/quality/version-management-committee/translation-process/.

[24] Stroke Association: Communication support pack. 2025 10/10/2025]; Available from: https://www.stroke.org.uk/stroke/effects/aphasia/communication-tools.

[25] Patel N, et al. Developing a Conceptually Equivalent Type 2 Diabetes Risk Score for Indian Gujaratis in the UK. J Diabetes Res. 2016;2016:8107108. 27703985 10.1155/2016/8107108 PMC5040802

[26] Lidington E, Stiles M, Maudsley J, et al. Establishing a set of acceptable demographic questions for use in health research through public consultation. Res Involv Engagem. 2026;12:24. 10.1186/s40900-026-00836-1. 10.1186/s40900-026-00836-1 PMC12924441 41606646

[27] IMPACT ('IMplemented by Parents And Carers' Therapy) trial: A multicentre randomised controlled trial to evaluate the efficacy of a parent-implemented therapy on language development in deaf children with cochlear implants. Award ID: NIHR154749. 2024 Cited 2025 22/10/2025; Available from: https://fundingawards.nihr.ac.uk/award/NIHR154749.

[28] A randomised controlled trial evaluating the efficacy and mechanisms of online perinatal emotional skills groups compared to treatment as usual for women and birthing people with borderline personality disorder. Acronym: PROSPER: PeRinatal emotiOnal skillS Personality disordER. Award ID: NIHR167051. 2025 Cited 2025 22/10/2025; Available from: https://fundingawards.nihr.ac.uk/award/NIHR167051.

[29] Hasegawa Y, et al. Ethnic difference in hematological toxicity in patients with non-small cell lung cancer treated with chemotherapy: a pooled analysis on Asian versus non-Asian in phase II and III clinical trials. J Thorac Oncol. 2011;6(11):1881–8. 21841503 10.1097/JTO.0b013e31822722b6

[30] Li LJ, et al. Different treatment efficacies and side effects of cytotoxic chemotherapy. J Thorac Dis. 2020;12(7):3785–95. 32802458 10.21037/jtd.2019.08.63 PMC7399437

[31] Divi C, et al. Language proficiency and adverse events in US hospitals: a pilot study. Int J Qual Health Care. 2007;19(2):60–7. 17277013 10.1093/intqhc/mzl069

[32] Charles A, et al. Proportionate translation of study materials and measures in a multinational global health trial: methodology development and implementation. BMJ Open. 2022;12(1):e058083. 35058270 10.1136/bmjopen-2021-058083 PMC8783829

[33] Eremenco S, et al. Patient-reported outcome (PRO) consortium translation process: consensus development of updated best practices. J Patient-Reported Outcomes. 2017;2(1):12. 10.1186/s41687-018-0037-6 PMC5934912 29757299

[34] Bamford J, et al. Falling on deaf ears: interpreters as cultural brokers in mental healthcare. BJPsych Bull. 2024;48(2):73–7. 38178800 10.1192/bjb.2023.90 PMC10985732

[35] Flores G, et al. Errors in medical interpretation and their potential clinical consequences in pediatric encounters. Pediatrics. 2003;111(1):6–14. 12509547 10.1542/peds.111.1.6

[36] Bolden GB. Toward understanding practices of medical interpreting: interpreters’ involvement in history taking. Discourse Stud. 2000;2(4):387–419.

[37] Pardhan S, et al. Barriers and facilitators for engaging underrepresented ethnic minority populations in healthcare research: an umbrella review. Int J Equity Health. 2025;24(1):70. 40075407 10.1186/s12939-025-02431-4 PMC11905581

[38] Hillman SL, et al. Rates of and factors associated with patient withdrawal of consent in cancer clinical trials. JAMA Oncol. 2023;9(8):1041–7. 37347469 10.1001/jamaoncol.2023.1648 PMC10288373

[39] Slade AL, et al. Systematic review of the use of translated patient-reported outcome measures in cancer trials. Trials. 2021;22(1):306. 33902699 10.1186/s13063-021-05255-z PMC8074490

[40] Health Research Authority. Publication and dissemination of research findings. 2023 Cited 2025 22/10/2025; Available from: https://www.hra.nhs.uk/planning-and-improving-research/best-practice/publication-and-dissemination-research-findings/.

[41] South A, et al. Sharing clinical trial results with participants: important, expected and possible. Trials. 2025;26(1):107. 40140937 10.1186/s13063-025-08802-0 PMC11948716

[42] Health Research Authority. Offering to share a summary of results with participants. 2025 Cited 2025 22/10/2025; Available from: https://www.hra.nhs.uk/planning-and-improving-research/policies-standards-legislation/clinical-trials-investigational-medicinal-products-ctimps/clinical-trial-regulations-reform/guidance-on-changes-to-the-clinical-trials-regulations/research-transparency-requirements-for-clinical-trials/offering-to-share-a-summary-of-results-with-participants/.

[43] Treweek S, et al. How should trial teams make decisions about the proportions and diversity of the ethnic groups in their trial? Trials. 2024;25(1):768. 39543747 10.1186/s13063-024-08625-5 PMC11566274

[44] Treweek S, et al. Developing the INCLUDE ethnicity framework—a tool to help trialists design trials that better reflect the communities they serve. Trials. 2021;22(1):337. 33971916 10.1186/s13063-021-05276-8 PMC8108025

[45] Office for National Statistics: Ethnicity facts and figures: English language skills. 2018 26/03/2024 22/10/2025]; Available from: https://www.ethnicity-facts-figures.service.gov.uk/uk-population-by-ethnicity/demographics/english-language-skills/latest/#by-ethnicity-and-sex.

[46] Health Research Authority. Integrated Research Application System (IRAS). 2025 Cited 2025 22/10/2025; Available from: https://www.myresearchproject.org.uk.

[47] IRAS Help - Reference - Collated guidance -NHS REC. 2017 Cited 22/10/2025 2025; Available from: https://www.myresearchproject.org.uk/help/hlpcollatedqsg-nhsrec.aspx#629.

[48] Health Research Authority queries line, personal correspondence R. Lewis. Health Research Authority. 2023.

[49] Willis A, Isaacs T, Khunti K. Improving diversity in research and trial participation: the challenges of language. Lancet Public Health. 2021;6(7):e445–6. 34174997 10.1016/S2468-2667(21)00100-6

[50] National Voices: Community Languages Translation and Interpreting Services. 2024. https://www.nationalvoices.org.uk/publication/community-languages-translation-and-interpreting-services/. Accessed 8 Nov 2025.

[51] Karliner LS, et al. Language Access Systems Improvement initiative: impact on professional interpreter utilisation, a natural experiment. BMJ Open. 2024;14(1):e073486. 38176864 10.1136/bmjopen-2023-073486 PMC10773371

[52] Hoffman H, et al. Researcher Perceptions of Inclusion of Study Participants Who Use Languages Other Than English. JAMA Netw Open. 2025;8(3):e252380–e252380. 40152859 10.1001/jamanetworkopen.2025.2380 PMC11953757

[53] English Unlocked: How to work effectively with interpreters. Cited 2025 22/10/2025; Available from: https://www.englishunlocked.co.uk/learn-to-work-with-interpreters/.

[54] Centre for Ethnic Health Research: Cultural Competence. Cited 2025 23/10/2025; Available from: https://ethnichealthresearch.org.uk/training/cultural-competence/.

[55] Cheung K, et al. Patient information for cancer trials – a site survey of accessibility and translation requirements. International Clinical Trials Methodology Conference. 2024. Edinburgh. https://osf.io/ngb98/files/5frbz. Accessed 8 Nov 2025.

[56] do Carmo F. Full report: Evidence review of use of AI in the public sector. University of Surrey. 2025. 10.15126/901630.

[57] Guerreiro NM, et al. Hallucinations in large multilingual translation models. Trans Assoc Comput Linguist. 2023;11:1500–17.

[58] Synthesia. Synthesia- Turn text to video, in minutes. 2024 Cited 2024 8th July; Available from: https://www.synthesia.io/?r=0.

[59] The Northern Ireland Network for Trials Methodology Research: SWAT Store: Studies Within A Trial repository. Cited 2025 23/10/2025; Available from: https://www.qub.ac.uk/sites/TheNorthernIrelandNetworkforTrialsMethodologyResearch/SWATSWARInformation/Repositories/SWATStore/.

[60] Research Organization Registry (ROR) [Online database/registry]. 23/10/2025]; Available from: https://ror-hub.org/.

[61] Oxford Clinical Trials Research Unit: The Explain Initiative – Explainer Animations. 23/10/2025]; Available from: https://explain.octru.ox.ac.uk/.

[62] Wylde V, et al. Recommendations for developing accessible patient information leaflets for clinical trials to address English language literacy as a barrier to research participation. Trials. 2024;25(1):624. 39334243 10.1186/s13063-024-08471-5 PMC11430508

[63] MAPLE: Making trials more accessible through better patient information leaflets. University of Bristol. 23/10/2025]; Available from: https://maple.bristol.ac.uk/.

[64] Treweek S, et al. Who is in your trial? Improving the reporting of participant characteristics in trial protocols and results. Trials. 2025;26(1):338. 40926250 10.1186/s13063-025-08938-z PMC12421759

[65] Evaluation of translation and INterpretation serVIces implemented by clinical Trials units (CTUs) to increase recruitment of Ethnic minority groups in pragmatic randomised controlled trials (The INVITE study). Award ID: NIHR206906. 2025 23/10/2025]; Available from: https://fundingawards.nihr.ac.uk/award/NIHR206906.

